# Associations between Caregiver-Provider Communication Quality and Unmet Care Needs Among Older Adults

**DOI:** 10.21203/rs.3.rs-7761669/v1

**Published:** 2025-12-02

**Authors:** Jiaming Liang, Rafael Samper-Ternent, Brian Downer, Zhigang Xie

**Affiliations:** The University of Texas Health Science Center at Houston; The University of Texas Health Science Center at Houston; University of Texas Medical Branch; University of Florida

**Keywords:** caregiver, communication quality, healthcare provider, unmet care needs, older adult

## Abstract

**Background:**

Unmet care needs (lack of assistance with daily activities) among older adults are common and linked to adverse health outcomes, greater healthcare utilization, and caregiver burden. While effective patient-provider communication is known to improve care quality, less is understood about the role of caregiver-provider communication, particularly across sociodemographic, health, and caregiving contexts.

**Methods:**

We conducted a cross-sectional secondary analysis of pooled 2021–2023 National Health and Aging Trends Study (NHATS) and National Study of Caregiving (NSOC) data, restricted to community-dwelling older adults (65 + years) who received assistance from caregivers due to difficulty with daily activities, and had at least 1 caregiver who communicated with medical providers in the past year. The unit of analysis was the caregiver-older adult pair, with clustering at the older adult level. The outcome was unmet care needs (0, 1, or 2+, across 12 daily activities). The primary predictor was caregiver-reported provider communication quality (range: 3–12). Weighted ordered logistic regression estimated associations between communication quality and unmet care needs, adjusting for older adult and caregiver sociodemographic, health, and caregiving characteristics.

**Results:**

The analytic sample included 1,414 older adults (weighted N = 9.34M) and 1,910 caregivers (weighted N = 21.05M). Half (50.5%) of older adults reported no unmet care needs, 23.5% reported one, and 26.0% reported 2 or more. Higher communication quality was associated with lower odds of greater unmet needs (OR = 0.93, 95%CI: 0.89–0.97). The association was stronger for low-income households (<$30K/year), for adult child caregivers and other relatives compared with spouses, and for caregivers providing < 20 hours or > 40 hours/month of care.

**Conclusions:**

Improving caregiver-provider communication may be an effective strategy for reducing unmet care needs among older adults with long-term care needs. Targeted efforts might be particularly needed for low-income families and non-spousal caregivers, but strategies must address systemic and logistical barriers to ensure equitable benefit.

## INTRODUCTION

The United States is experiencing a major demographic shift: the population aged 65 + is growing rapidly due to the aging Baby Boomers and increased longevity.[1] Many older adults prefer to age in place, about one in four report difficulty with at least one activity of daily living (ADL) or instrumental activity of daily living (IADL), and nearly half receive assistance from family or other caregivers because of chronic illness, functional limitations, or disability.[2, 3] Despite this support, unmet care needs (i.e., lack of needed help with daily self-care, mobility, or household activities) remain common, and are linked to poorer health, greater healthcare use, premature institutionalization, and caregiver stress.[4, 5] Therefore, identifying modifiable factors, such as effective caregiver-provider communication, that reduce unmet care needs among community-dwelling older adults is essential for guiding clinical, community, and policy strategies.

Unmet care needs affect roughly 25% of older adults.[6] Among those with dementia or functional limitations, at least one in five (20%) receive no personal assistance for basic or instrumental daily activities, and these gaps are more common among underrepresented racial/ethnic groups, individuals with lower socioeconomic status, and those with complex health needs.[7] Unmet needs are linked to functional decline,[8] falls,[9] hospitalizations,[10] premature nursing home placement,[11] and increased caregiver burden and family strain.[12] Although often under-recognized in clinical assessments, addressing unmet needs is now a public health priority, as the National Institute of Health (NIH) recently designated people with disabilities, including those facing unmet care needs, as a health-disparities population warranting targeted research and interventions.[13]

Effective communication between patients and healthcare providers, such as careful listening and clear explanation of care plans, is consistently associated with improved treatment adherence, chronic disease management, patient satisfaction, and overall care experiences.[14–16] Conversely, poor patient-provider communication can result in misunderstandings about care plans, reduced engagement, and unmet needs, compromising quality of care. In contrast, caregiver-provider communication has received comparatively less empirical attention. Emerging evidence indicates that timely, transparent exchanges between providers and family caregivers can reduce caregiver stress, enhance service satisfaction, and promote care coordination.[17] Such communication may help meet older adults’ long-term care needs by clarifying specific support requirements, empowering caregivers to navigate healthcare systems, and facilitating timely referrals to appropriate services. Prior studies also show that when caregivers connect to supportive services, such as respite care, care coordination programs, and home- and community-based services, older adults are more likely to have their daily care needs met and experience fewer service gaps.[18, 19]

Additionally, the impact of caregiver-provider communication on unmet care needs may vary across sociodemographic, health, and caregiving contexts. Factors such as age, sex, education, race/ethnicity, and household income can shape both communication experiences and access to services.[20–22] Similarly, the health status of older adults, their relationship with their caregivers (e.g., spouse, non-family member), care intensity, and living arrangements may also influence how communication leads to timely service use, adherence to care plans, and reduced unmet needs. Considering these factors can help identify subgroups of older adults most likely to benefit from improved caregiver-provider communication.

This study examines the association between caregiver-provider communication quality and unmet care needs among community-dwelling older adults with long-term care needs, using nationally representative data. We hypothesized that higher-quality communication will be associated with fewer unmet care needs. We also explored whether this relationship varies by key contextual factors, including older adults’ sociodemographic characteristics (age, sex, race/ethnicity, marital status, and annual household income), health status (self-rated health, functional limitations, and dementia status), and caregiving characteristics (caregiver’s age, sex, relationship to the older adult, and caregiving intensity). Findings from this study will enhance understanding of the mechanisms through which the quality-of-care caregivers provide changes and can help identify caregivers who may benefit most from targeted communication-promotion interventions.

## METHODS

### Study design and data source

This cross-sectional study examined the association between caregiver-provider communication quality and unmet care needs among community-dwelling older adults in the United States. The analytic sample included older adults and their family or unpaid caregivers. Because some older adults received help from multiple caregivers (ranging from 1 to 5), the unit of analysis was the caregiver-older adult pair, with analytic procedures clustering multiple caregivers within each older adult.

Data were drawn from the National Health and Aging Trends Study (NHATS) and its companion, the National Study of Caregiving (NSOC).[23] NHATS is a nationally representative, longitudinal survey of Medicare beneficiaries aged 65+, launched in 2011, with annual interviews, periodic sample replenishment, and oversampling of Black and Hispanic older adults. NSOC, initiated in 2011 and conducted periodically and annually since 2021, links to NHATS respondents and surveys eligible family and unpaid caregivers reported by each older adult. NSOC collects detailed data on caregiving experience and its effects on caregiver health and well-being. Together, NHATS and NSOC provide the only national resource for simultaneously studying care recipient and caregiver perspectives. The NHATS and NSOC were approved by the Institutional Review Board at the Johns Hopkins Bloomberg School of Public Health, and all participants provided informed consent at enrollment. The data for this study are de-identified and publicly available. The institutional review board at the first author’s university determined that this study did not involve human subjects and was exempt from review.

### Sample selection

Eligibility for this study was limited to community-dwelling older adults who (1) received assistance from a family or unpaid caregiver with daily activities in the past year, (2) had ≥ 1 caregiver identified and interviewed in NSOC, and (3) had ≥ 1 interviewed caregiver who reported communicating with medical providers in the past year. These criteria align with the research focus, on how caregiver-provider communication relates to unmet care needs, and ensure that both caregiver characteristics and communication quality can be measured. The analytic sample pooled non-overlapping records from NHATS Rounds 11–13 (2021–2023), ensuring measurement consistency for communication variables across waves and leveraging the post-COVID sample replenishments (Rounds 12–13), which enhanced representation of Black and Hispanic older adults. This approach yielded a contemporary, diverse, and well-characterized sample.

From the pooled, non-overlapping sample across three waves, 2,439 older adults and 3,673 caregivers were identified. After applying the eligibility criteria, the final analytic sample included 1,414 (weighted N = 9,338,122) older adults and 1,910 (weighted N = 21,047,219) caregivers (by wave: 441, 393, and 580 older adults in Rounds 11, 12, and 13, respectively).[24] Detailed comparisons of older adults with and without caregiver-provider communication are provided in Supplementary Table S1. Those included in the analytic sample tended to have poorer health, as reflected in a higher prevalence of functional limitations (48.3% vs. 36.7%; χ^2^=31.3, p < .001). To minimize potential confounding due to the high correlation between greater functional limitations and reported unmet care needs, we excluded older adults whose caregivers did not communicate with providers, as these individuals were less likely to report unmet care needs due to better health. Missing data on key variables (e.g., communication frequency and quality) were minimal (2.7%) and excluded from analysis.

### Measures

#### Outcome: unmet care needs of older adults

In NHATS, unmet care needs are defined as negative consequences due to activity difficulty when no one provides assistance.[25] Respondents reported (yes/no) whether, in the past 12 months, they experienced any of 12 consequences across self-care, mobility, and household domains: going without eating; without showering, bathing, or washing up; accidentally wetting or soiling clothes; getting dressed; having to stay in bed; not being able to move about the home or building; being unable to leave the home or building; without clean clothes; without groceries or personal items; without a hot meal; without help with bills or banking; and making a mistake in taking medications. We summed consequences to create a total score (range: 0–12). For analytic purposes, we categorized unmet care needs of older adults as 0, 1, or 2 or more, to address the skewed distribution (excess of 0s) and facilitate interpretation in regression models.

#### Key predictor: caregiver-provider communication quality

NSOC asked three follow-up questions of caregivers who reported interacting with medical providers regarding the care recipient in the past year: “In the last year, how often did that provider (a) listen to what you had to say; (b) ask if you understood the care recipient’s health treatments; and (c) ask if you needed help managing the care recipient’s health treatments?” Responses ranged from 1 = never to 4 = always. Although prior studies used these items individually,[24, 26] we combined them to capture caregivers’ overall perception of communication quality, and the three items demonstrated good internal consistency (Cronbach’s α = 0.68). The final composite score ranges 3–12, with higher scores indicating better perceived communication quality between caregivers and healthcare providers.

#### Covariates

Informed by prior research and our analytic framework, models adjusted for older-adult and caregiver characteristics to address confounding and assess effect modification. For older adults, we included: age (79 or younger/80–89/90 or older), sex (female/male), race and ethnicity (Non-Hispanic White/Non-Hispanic Black/Hispanic/Other), marital status (married/not married), annual household income (<$30,000/$30,000-$70,000, >$70,000), self-rated health (good or better/fair or poor), number of functional limitations (self-reported having difficulty completing by self, range: 0–12), and dementia status (yes/no).[27] Caregiver-level factors include: age (18–54/55–64/65–74/75 or older), sex (female/male), relationship to care recipient (spouse/adult child/other relative or non-relative), size of care network (number of caregivers providing assistance: 1/2/3+), care intensity (hours of care provided per month: <20/20–40/>40), and co-residence with the older adult (yes/no).

#### Analytic strategies

All analyses were conducted using Stata SE 18.[28] We first conducted descriptive analyses to characterize the sociodemographic, health, and caregiving characteristics of the analytic sample. Variable distributions were summarized as frequencies and percentages for categorical variables and as means and standard errors for continuous variables. Potential collinearity among predictors and covariates were examined through pairwise correlations and variance inflation factors (all VIFs < 2.0). The primary analytic model used ordered logistic regression to examine the association between caregiver-provider communication quality scores and older adults’ unmet care needs (proportional odds assumption met, Brant test p > 0.05). To further explore effect modification, a series of interaction models were estimated, each including product terms between communication quality scores and sociodemographic (e.g., age, race/ethnicity, income) or caregiving characteristics (e.g., relationship type, care intensity). Improvements in model fit following the inclusion of interaction terms were evaluated using likelihood ratio tests and changes in information criteria. Significant interactions were subsequently visualized to facilitate interpretation.

All analyses accounted for the complex survey design of NHATS and NSOC by incorporating the final analytic weights, which generate nationally representative estimates for older adults and their caregivers who communicated with healthcare providers.[29] To address the presence of multiple caregivers per older adult, reflecting the nested data structure, standard errors were adjusted for clustering at the older adult level. Several sensitivity analyses were conducted to assess the robustness of findings. Alternative model specifications included restricting analyses to primary caregivers only, selecting the most frequent caregiver-provider communicator, and adjusting for primary caregiver status and the size of the caregiver network. Additionally, we estimated models using zero-inflated negative binomial regression to compare with the findings and model fit indices from the primary analytic models to ensure consistency and reliability of analyses.

## RESULTS

### Sample description

The analytic sample (Table 1) included 1,414 community-dwelling older adults (weighted N = 9,338,122) and 1,910 family or unpaid caregivers (weighted N = 21,047,219). Among older adults, 58.7% were aged 79 or younger, 57.8% were female, and 70.5% were non-Hispanic White, with 11.9% non-Hispanic Black and 12.5% Hispanic. Over half (53.0%) were married, one-third (32.9%) reported an annual household income below $30,000, and half (50.6%) rated their health as good or better. The weighted mean number of functional limitations was 6.2, and 22.3% had possible/probable dementia. Among caregivers, 68.6% were female, 53.1% were adult children, and 29.0% were spouses. Over half (54.2%) provided more than 40 hours of care per month, and 55.5% lived with the older adult.

Regarding caregiver-provider communication quality, the weighted mean score was 8.8 (range: 3–12). For unmet care needs of older adults, 50.5% of older adults reported none, 23.5% reported one, and 26.0% reported two or more unmet care needs. Most older adults (68.5%) had a single caregiver, 24.7% had two caregivers, and 6.8% had three or more. Since we only included older adults who have at least one caregiver who talked to providers, we found that the majority of care networks (if an older adult has multiple caregivers) had all caregivers interact with providers; in networks with two or more caregivers, on average, 66.4% of caregivers reported having provider communication in the last year (Supplementary Table S2).

### Association between caregiver-provider communication quality and unmet care needs of older adults

In the primary weighted ordinal logistic regression model (Table 2), higher caregiver-provider communication quality was significantly associated with lower odds of greater unmet care needs among older adults (OR = 0.93, 95% CI: 0.89–0.97, p = 0.001). Among the adjusted covariates, older adults’ age, race/ethnicity, self-rated health, number of functional limitations, and co-residential status with caregivers were significantly associated with unmet care needs. Specifically, compared to those aged 79 or younger, adults aged 80–89 (OR = 0.70, 95% CI: 0.53–0.93, p = 0.01) and those aged 90 and older (OR = 0.62, 95% CI: 0.43–0.90, p = 0.01) had lower odds of unmet care needs. Non-Hispanic Black older adults reported lower odds than their non-Hispanic White counterparts (OR = 0.74, 95% CI: 0.55–0.98, p = 0.04). Better self-rated health was also associated with reduced odds (OR = 0.71, 95% CI: 0.57–0.90, p = 0.004), while a higher number of functional limitations increased the odds (OR = 1.46, 95% CI: 1.40–1.53, p < 0.001). Having a caregiver who resided in the same household was associated with lower odds of unmet care needs (OR = 0.63, 95% CI: 0.48–0.83, p = 0.001).

### The moderation effects of income, relationship type, and caregiving intensity

Significant moderation effects were found for family income level, caregiver relationship type, and caregiving intensity (hours per month). Full model results for these interactions are presented in Supplementary Table S3.

**Figure 1:**
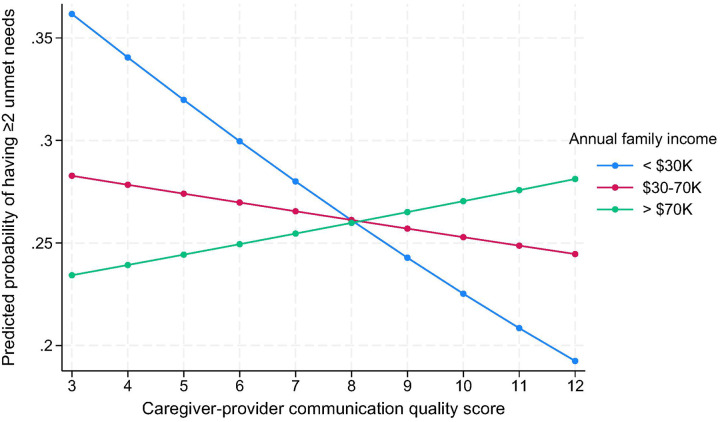
Predicted probability of having ≥2 unmet needs by caregiver-provider communication and annual income. Figure 1 shows predicted probabilities of reporting two or more unmet care needs among community-dwelling older adults by caregiver-provider communication quality scores, stratified by household income level (<$30,000; $30,000–$70,000; >$70,000). Stronger protective effects of higher communication quality were observed among older adults in low-income households.

The association between higher caregiver-provider communication quality and unmet care needs was significantly stronger among older adults from low-income households (<$30K/year), while the association was attenuated in those with annual incomes of $30–70K (OR = 1.17, 95% CI: 1.01–1.35, p = 0.03) and >$70K (OR = 1.24, 95% CI: 1.04–1.48, p = 0.02), as shown in Fig. 1.

**Figure 2:**
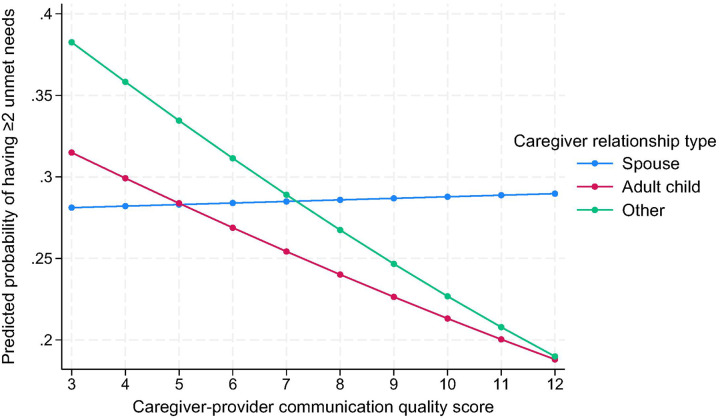
Predicted probability of having ≥2 unmet needs by caregiver-provider communication and caregiver relationship type Figure 2 illustrates predicted probabilities of reporting two or more unmet care needs among older adults by caregiver-provider communication quality scores, stratified by caregiver relationship type (spouse, adult child, other relative or non-relative). The association between higher communication quality and lower unmet care needs was most pronounced for adult children and other relatives, compared with spousal caregivers.

Regarding caregiver relationship, the protective association of higher communication quality with unmet care needs was stronger when care was provided by adult children (OR = 0.90, 95% CI: 0.82–0.99, p = 0.04) or others (OR = 0.85, 95% CI: 0.72–0.99, p = 0.04), compared to spouses (Fig. 2).

**Figure 3:**
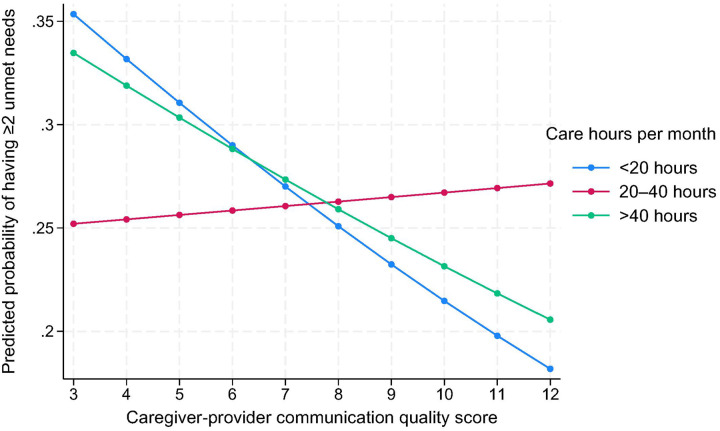
Predicted probability of having ≥2 unmet needs by caregiver-provider communication and caregiving hours per month Figure 3 presents predicted probabilities of reporting two or more unmet care needs by caregiver-provider communication quality scores, stratified by caregiving intensity (<20 hours, 20–40 hours, >40 hours per month). The protective effect of high-quality communication was strongest at low and high caregiving intensity, while the effect was attenuated at moderate caregiving intensity (20–40 hours/month).

For caregiving intensity, a significant interaction was observed: the negative association between communication quality and unmet care needs was strongest among caregivers providing < 20 or > 40 hours/month, but was attenuated at 20–40 hours/month (OR = 1.15, 95% CI: 1.02–1.31, p = 0.03), as illustrated in Fig. 3.

## DISCUSSION

This study examined whether higher caregiver-provider communication quality was associated with fewer unmet care needs among community-dwelling older adults with long-term care needs. Consistent with our hypothesis, better communication was linked to a lower likelihood of unmet needs, even after accounting for sociodemographic, health, and caregiving factors. Importantly, the association was strongest in low-income households, more pronounced for adult children and other relatives than for spouses, and attenuated at moderate caregiving intensity (20–40 hours/month). These patterns suggest that systematically engaging caregivers, during clinic visits, discharge planning, and home health assessments may be particularly effective for identifying overlooked needs and coordinating timely support, thereby potentially improving care quality and outcomes for older adults.

Within our analytic sample, caregiver involvement in communication with providers was common. Among older adults with two or more caregivers, an average of 66% of caregivers in each care network had contacted providers in the past year. We restricted the sample to older adults with at least one caregiver who had communicated with providers, thereby avoiding confounding from healthier individuals who did not require caregiver involvement in medical care coordination.[26, 30] This focused sample therefore enabled a more precise examination of our research question. Given the central role caregivers play in relaying information, advocating for care, and facilitating follow-up, the quality of these interactions is likely a key determinant of whether older adults’ needs are fully identified and addressed.

Our finding that higher caregiver-provider communication quality was associated with fewer unmet care needs among older care recipients advances understanding of the dynamic roles of older adults, providers, and caregivers in this triadic relationship. Prior evidence demonstrates that patient-provider communication positively influences service satisfaction, physical health outcomes, mental health, and overall well-being, yet the caregiver’s role within this context has received little attention. Communication is a fundamental driver of care quality, and caregivers are uniquely positioned to overcome many barriers that can hinder exchanges between patients and providers, such as seeking clarification of key information and diagnoses, understanding and organizing treatment plans, and relaying them to the patient with empathy.[31–34] In practice, these contributions can improve the accuracy and completeness of needs assessments, foster shared understanding of care plans, and connect families to appropriate resources in a timely manner. The significant finding in our nationally representative sample suggests that enhancing caregiver engagement is not a marginal improvement but rather a potentially high-yield strategy for reducing unmet needs in routine care.

In addition, the association between caregiver-provider communication quality and unmet care needs among older adults was not uniform, with stronger effects observed among low-income households, adult children or other relative caregivers compared to spouses, and those providing either low (< 20 hours/month) or high (> 40 hours/month) intensity care. In low-income families, limited resources, lower health literacy, and barriers to accessing care heighten reliance on providers for clear, actionable information to navigate the healthcare system and address care needs.[35, 36] Adult children and other relatives, who may live apart, have less day-to-day contact and face greater social, emotional, and financial burdens, depend heavily on providers for guidance, updates, and coordination.[21, 37, 38] For caregivers providing minimal hours, strong communication ensures they remain well-informed and are able to detect emerging needs, while for those with high care intensity, it can help manage complex care demands, prevent errors, and reduce burnout.[39–41] Although understanding of these variances is limited, these findings suggest that targeted efforts to strengthen caregiver-provider communication in specific subgroups could reduce unmet needs and improve care quality for older adults.

### Implications

This study has important implications for practice and policy. Interventions that strengthen caregiver-provider communication, particularly through communication skills training, can improve care for older adults and support caregiver well-being. Such training equips caregivers to clearly convey observations and concerns, facilitating accurate needs identification, care coordination, and timely service access, while reducing confusion and errors. It also builds caregivers’ confidence in initiating discussions, asking questions, and addressing sensitive topics such as advance care planning, thereby reducing anxiety and distress.[42–44] Evidence suggests these benefits might be particularly valuable for low-income caregivers, who may face greater barriers to care, and for non-spousal caregivers, such as adult children, who often have less direct caregiving experience.[45]

Clinically, providers should proactively include caregivers in discussions, use plain language, confirm understanding, and offer multiple communication modalities to accommodate geographic distance and work constraints.[19] Moreover, tailoring communication to the specific disease context and caregiver type may enhance its effectiveness, reduce unmet needs, and ultimately improve health outcomes for both patients and caregivers.[46] Health systems should integrate caregiver information into electronic health records, develop standardized engagement protocols, and incentivize provider-caregiver communication through quality metrics and reimbursement models.[47] Embedding caregiver-provider communication as a critical element of patient- and family-centered care can simultaneously improve older adults’ outcomes, reduce caregiver burden, and promote equity and sustainability in long-term care delivery.

### Limitations

This study has several limitations. First, because of its cross-sectional design, we cannot conclude that caregiver-provider communication quality has a causal influence on reducing unmet care needs; longitudinal research is needed for further verification. Second, caregiver-provider communication quality was self-reported and measured using only three items, which may introduce bias and fail to capture important dimensions such as cultural competence and specific aspects of care coordination. It is necessary to develop and validate a structured instrument to more comprehensively assess caregiver-provider communication quality in future studies.[48] Third, and important for generalizability, the analytic sample included only older adults with at least one caregiver who had contacted providers in the past year, potentially excluding those with substantial medical needs but no caregiver involvement, who may be especially vulnerable. Although we examined interactions with older adults’ health status (self-rated health, dementia, functional limitations) and found no effect modification, future research should incorporate condition-specific measures (e.g., cancer, advanced heart failure) to better understand subgroup differences.

## CONCLUSION

Caregiver-provider communication is a critical yet underutilized pathway for identifying and addressing older adults’ unmet care needs. Enhancing this communication, particularly for vulnerable subgroups such as low-income and non-spousal caregivers, has the potential to improve care quality, reduce caregiver burden, and promote equitable, person- and family-centered care. Realizing these benefits will require integrating caregivers systematically into clinical workflows, adopting validated tools to assess communication quality, and implementing policies that incentivize and support meaningful caregiver engagement across healthcare settings.

## Supplementary Material

Supplementary Files

This is a list of supplementary files associated with this preprint. Click to download.

- Additionalfile.pdf

## Figures and Tables

**Table 1:** Sample descriptions

Variable	Category	N (weighted %) / [weighted Mean (SE)]
Older adults (N = 1414; weighted N = 9,338,122)		
Age	79 or younger	576 (58.72%)
	80–89	554 (30.37%)
	90 or older	284 (10.91%)
Sex (female)		889 (57.76%)
Race and ethnicity	Non-Hispanic White	728 (70.52%)
	Non-Hispanic Black	346 (11.88%)
	Hispanic	279 (12.47%)
	Other	61 (5.13%)
Marital status (married)		603 (52.95%)
Annual household income	< 30K USD	577 (32.92%)
	30–70K USD	432 (33.17%)
	> 70K USD	404 (33.91%)
Self-rated health (good or better)		703 (50.63%)
Number of functional limitations [range: 0–12]		[6.24 (0.12)]
Dementia status (yes)		377 (22.34%)
Unmet care needs	0	728 (50.52%)
	1	347 (23.53%)
	2 or more	339 (25.95%)
Caregivers (N = 1910; weighted N = 21,047,219)		
Age	18–54	525 (34.68%)
	55–64	538 (25.54%)
	65–74	481 (26.38%)
	75 or older	295 (13.40%)
Sex (female)		1409 (68.63%)
Relationship type	Spouse	507 (29.04%)
	Adult child	1133 (53.10%)
	Other	270 (17.86%)
Size of care network	1	877 (68.46%)
	2	408 (24.73%)
	3 or more	129 (6.81%)
Care intensity (hours/month)	< 20	440 (23.94%)
	20–40	402 (21.85%)
	> 40	1068 (54.21%)
Living with older adults		1068 (55.45%)
Caregiver-provider communication quality [range: 3–12]		[8.82 (0.08)]

**Table 2.** Ordinal logistic regression of caregiver-provider communication quality and unmet care needs among older adults (N = 1,414)

Variable	Category	OR (95% CI)
Caregiver-provider communication quality		0.93 (0.89, 0.97)**
Older adult age (ref: 79 or younger)	80–89	0.70 (0.53, 0.93)*
	90 or older	0.62 (0.43, 0.90)*
Older adult sex (female vs. male)		1.30 (1.00, 1.68)
Race/ethnicity (ref: Non-Hispanic White)	Non-Hispanic Black	0.74 (0.55, 0.98)*
	Hispanic	1.10 (0.79, 1.53)
	Other	0.79 (0.48, 1.30)
Household income (ref: <$30K)	$30–70K	1.10 (0.77, 1.56)
	>$70K	1.20 (0.73, 1.97)
Self-rated health (good or better vs. fair or poor)		0.71 (0.57, 0.90)**
Number of functional limitations		1.46 (1.40, 1.53)***
Dementia status (yes vs. no)		0.82 (0.62, 1.08)
Caregiver age (ref: 18–54 years)	55–64	0.89 (0.65, 1.21)
	65–74	0.81 (0.56, 1.17)
	75 or older	0.66 (0.42, 1.06)
Caregiver sex (female vs. male)		0.84 (0.66, 1.08)
Caregiver relationship (ref: Spouse)	Adult child	0.79 (0.50, 1.25)
	Other	0.99 (0.59, 1.67)
Caregiving hours per month (ref: <20 hours/month)	20–40 hours/month	1.17 (0.85, 1.61)
	< 40 hours/month	1.06 (0.79, 1.40)
Coresident caregiver (yes vs. no)		0.63 (0.48, 0.83)**

*Notes:* Odds ratios (OR) are weighted estimates. Robust standard errors were used.

* p < 0.05,

** p <0.01,

*** p <0.001.

## Data Availability

This study used publicly available secondary data from the National Health and Aging Trends Study (NHATS) and the National Study of Caregiving (NSOC). https://nhats.org

## Abbreviations

NHATS: National Health and Aging Trends Study
NSOC: National Study of Caregiving
ADL: Activities of Daily Living
IADL: Instrumental Activities of Daily Living
